# Fludeoxyglucose positron emission tomography-computed tomography scan showing polyarthritis in a patient with an atypical presentation of Henoch-Schönlein vasculitis without clinical signs of arthritis: a case report

**DOI:** 10.1186/s13256-016-0913-8

**Published:** 2016-06-02

**Authors:** Christiaan F. Mooij, Rick Hermsen, Esther P. A. H. Hoppenreijs, Chantal P. Bleeker-Rovers, Marloes M. IJland, Lioe-Fee de Geus-Oei

**Affiliations:** 1 Department of Pediatrics, Amalia Children’s Hospital, Radboud University Medical Centre, PO Box 9101, 6500 HB Nijmegen, The Netherlands; 2 Department of Radiology and Nuclear Medicine, Radboud University Medical Centre, PO Box 9101, 6500 HB Nijmegen, The Netherlands; 3 Department of Pediatric Rheumatology, Amalia Children’s Hospital, Radboud University Medical Centre, PO Box 9101, 6500 HB Nijmegen, The Netherlands; 4 Department of Internal Medicine, Radboud University Medical Centre, PO Box 9101, 6500 HB Nijmegen, The Netherlands

**Keywords:** Henoch-Schönlein, FDG-PET/CT, polyarthritis

## Abstract

**Background:**

Henoch-Schönlein vasculitis is the most common systemic vasculitis in children. Arthritis or arthralgia occurs in 80 % of patients. We believe this to be the first case report to describe the finding of polyarthritis in a fludeoxyglucose positron emission tomography-computed tomography scan in a patient with Henoch-Schönlein vasculitis without clinical signs of arthritis.

**Case presentation:**

A 4.5-year-old Caucasian boy presented with fever of 4 days’ duration followed by debilitating migratory arthralgia and inflammation. He underwent a fludeoxyglucose positron emission tomography-computed tomography scan to exclude a possible malignant cause or to detect any infectious or autoimmune focus of his symptoms. Fludeoxyglucose uptake was observed in multiple large joints and in multiple tendons. These findings suggested active polyarthritis and polytendinitis. However, physical and ultrasound evaluations did not show any signs of arthritis in our patient, despite his evident arthralgia.

**Conclusions:**

Fludeoxyglucose positron emission tomography-computed tomography might be able to detect inflammatory activity in painful joints that cannot yet be detected clinically or with ultrasound evaluation in a patient with Henoch-Schönlein vasculitis. Therefore, fludeoxyglucose positron emission tomography-computed tomography can be of additional value in the diagnostic workup of patients with an unresolved diagnosis of suspected autoimmune disease, especially in patients with unresolved arthralgia and fever of unknown cause.

## Background

Henoch-Schönlein purpura is the most common systemic vasculitis in children. Henoch-Schönlein purpura is a type of small vessel vasculitis. Validated classification criteria for Henoch-Schönlein purpura have been proposed by the European League Against Rheumatism (EULAR) and Pediatric Rheumatology European Society (PRES). A patient’s disease is classified as Henoch-Schönlein purpura in the presence of purpura or petechiae (mandatory) with lower limb predominance plus one of four criteria: (1) abdominal pain; (2) histopathology (IgA); (3) arthritis or arthralgia; or (4) renal involvement [1].

The incidence of Henoch-Schönlein purpura is reported to be 10–20 per 100,000 children [2–6]. Henoch-Schönlein purpura can be diagnosed at any age, but is most common in children between 2 and 6 years of age. The rash of Henoch-Schönlein purpura is considered a classical symptom; however, in 30–40 % of cases it is not the presenting symptom, and the purpura is preceded by abdominal pain or arthralgia. Although only 15–25 % of patients with Henoch-Schönlein purpura present with arthritis or arthralgia, over the course of the disease approximately 80 % of patients have joint involvement [7]. The reported prevalence of arthritis as a symptom of Henoch-Schönlein purpura in pediatric patients varies in the literature from 61 to 92 % [8–13]. Usually the large joints of the lower limbs are affected. Symptoms of joint involvement include pain, swelling, and decreased range of movement [7].

In this report, we present the case of a 4.5-year-old boy with a final diagnosis of Henoch-Schönlein purpura who did not have clinical signs of arthritis. A fludeoxyglucose (FDG) PET/CT scan, however, showed FDG uptake in multiple large joints and in multiple tendons. ^18^F-FDG is a glucose analog that reflects the energy demand of inflammatory and/or malignant cells. Therefore, an increased uptake of ^18^F-FDG results from the increased metabolism of either inflammatory or malignant cells. In our case, the findings suggested active polyarthritis and polytendinitis, suggesting that an FDG-PET/CT scan can show inflammatory activity in painful joints that cannot yet be detected clinically or with ultrasound evaluation.

## Case presentation

A 4.5-year-old Caucasian boy without a previous medical history presented to the pediatric outpatient clinic of a local hospital with a fever of 40.1 °C without clear focal symptoms. He was reported as being less active. He refused to walk and reported mild myalgia, but did not complain about joint pain. His parents reported that he had had a painful throat 2–3 weeks prior to presentation. At that time, no doctor was consulted. Two weeks prior to presentation his parents noticed peeling of the skin on his fingers, which recurred 1 day prior to presentation. A timeline of this case is shown in Table 1. Our patient was vaccinated according to the Dutch National Immunization Program.

**Table 1:** A timeline of events for a 4.5-year-old boy with Henoch-Schönlein vasculitis

Time point	Event
Two week prior to presentation	• Upper respiratory tract infection with a painful throat, no doctor was consulted
Day 1 - Presentation	• Presentation to a local hospital with fever without clear focal symptoms • Treatment with amoxicillin-clavulanic acid for a suspected bacterial lymphadenopathy
Day 4	• Patient was referred to the university hospital because of possible Kawasaki disease
Day 7	• Treatment with intravenous immunoglobulins for possible incomplete Kawasaki disease
Day 8	• Treatment with intravenous immunoglobulins for possible incomplete Kawasaki disease • Migrating joint pains in his neck, arms, and legs • No signs of arthritis on physical examination
Day 9	• Started treatment with diclofenac
Day 13	• FDG-PET/CT: increased FDG uptake in multiple joints (polyarthritis) and multiple bilateral cervical lymph nodes
Day 14	• Ultrasound evaluation of his joints: no signs of arthritis
Day 17	• Clinical improvement with diclofenac treatment • Discharged without establishing a diagnosis
Day 24	• Evaluation at our outpatient clinic • Multiple purpura on his lower limbs and buttocks for 1 day • Skin biopsy: leukocytoclastic vasculitis with positive IgA depositions • Diagnosis was established: Henoch-Schönlein vasculitis • Treatment with diclofenac was continued
Day 25	• Presentation with bloody stools and abdominal pain • Ultrasound: no signs of invagination or thickened intestinal walls • Treatment with prednisolone was started, diclofenac was discontinued
Day 35	• Clinical improvement • Dosage of prednisolone was lowered
Day 63	• Relapse in joint pain and abdominal pain • Renal manifestation of Henoch-Schönlein vasculitis: hematuria and proteinuria • Dosage of prednisolone was increased
Day 73	• No clinical signs of Henoch-Schönlein vasculitis • Treatment with prednisolone was discontinued after gradually lowering the dosage

*FDG-PET/CT* fludeoxyglucose positron emission tomography-computed tomography, *IgA* immunoglobulin A

A physical examination at presentation revealed a left-sided torticollis and edema of both hands and feet. Our patient was irritable, without clinical sings of meningitis. The rest of his physical examination, including an inspection of his ears, nose, and throat, was normal. His vital signs were normal. An ultrasound evaluation demonstrated bilateral enlarged cervical lymph nodes, not noted on palpation during the physical examination. Because lymphadenopathy of a bacterial origin was suspected, treatment was started with an intravenous course of amoxicillin-clavulanic acid and diclofenac. A blood test revealed an anti-streptolysin O titer of 7590 U/ml, suggestive of a prior streptococcal infection. Despite this treatment, his fever persisted. Owing to the combination of fever, edema of hands and feet, mild conjunctivitis, cervical lymphadenopathy, and irritable behavior, Kawasaki disease was considered and he was referred to our university hospital for further evaluation 3 days after presentation.

On cardiac ultrasound evaluation, no involvement of his coronary arteries was observed. A biochemical evaluation showed an increased erythrocyte sedimentation rate (127 mm/h) and low CRP (9 mg/l). Although our patient did not meet the diagnostic criteria for Kawasaki disease, given that his fever had only persisted for up to 4 days, he was treated twice with 2 g/kg bodyweight of intravenous immunoglobulins (IVIG) for possible incomplete Kawasaki disease. Treatment with IVIG, however, did not result in clinical improvement. The enlargement of his cervical lymph nodes decreased over time as evaluated by ultrasonography.

Our patient then developed migrating joint pains in his neck, arms, and legs 8 days after presentation. Diclofenac, but not acetaminophen, was effective in treating these arthralgias. Diclofenac treatment (12.5 mg thrice daily) was started 9 days after presentation and his arthralgia resolved within 2 days of treatment. He also developed finger contractures: both active and passive straightening were affected. Furthermore, an antalgic gait was observed. He did not have any clinical signs of arthritis. During his hospital stay, our patient developed hypertension (highest blood pressure 130/93 mmHg; above the *p90* for sex and age). Because he did not improve clinically over time and no infectious cause could be identified, an autoimmune disease or a malignancy with paraneoplastic arthralgias was considered. Results of laboratory investigations are shown in Table 2. To exclude a possible malignant cause or to detect any infectious or autoimmune focus, an FDG-PET/CT scan was performed.

**Table 2:** Results of laboratory tests

Laboratory test	Result	Time of laboratory test
Hemoglobin	6.0 mmol/l	Day 6
Leukocytes	10.9 × 10^9^/l	Day 6
Manual blood count	No abnormalities	Day 6
Alanine aminotransferase (ALAT)	29 U/l	Day 6
Aspartate aminotransferase (ASAT)	42 U/l	Day 6
Gamma-glutamyl transpeptidase	14 U/l	Day 6
Lactate dehydrogenase (LDH)	370 U/l	Day 6
Creatinine	25 μmol/l	Day 6
Urea	5.7 mmol/l	Day 6
Ferritin	100 μg/l	Day 13
Anti-Nuclear Antibody (ANA)	Weak positive	Day 9
Anti-Neutrophil Cytoplasmic Antibody (ANCA)	Negative	Day 9
IgG	28.80 g/l	Day 7
Urine test	No proteinuria and no hematuria	Day 4, 9, 12, and 24

**Fig. 1:**
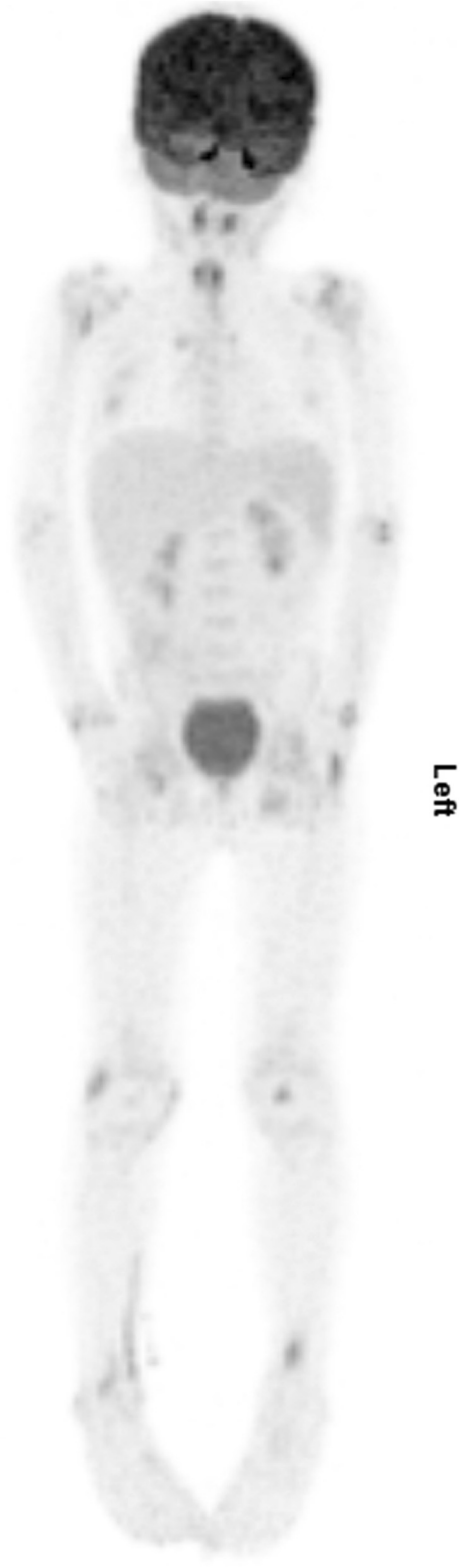
Maximum intensity projection of the patient. The projection shows increased fludeoxyglucose uptake in multiple joints and at the site of injection in the right lower leg

**Fig. 2:**
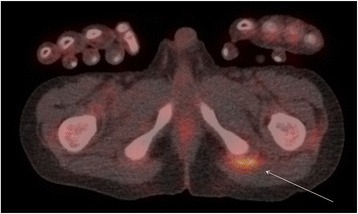
Detailed image of fludeoxyglucose positron emission tomography-computed tomography scan showing increased fludeoxyglucose uptake in the left ischiopubic tendon attachments (*arrow*)

**Fig. 3:**
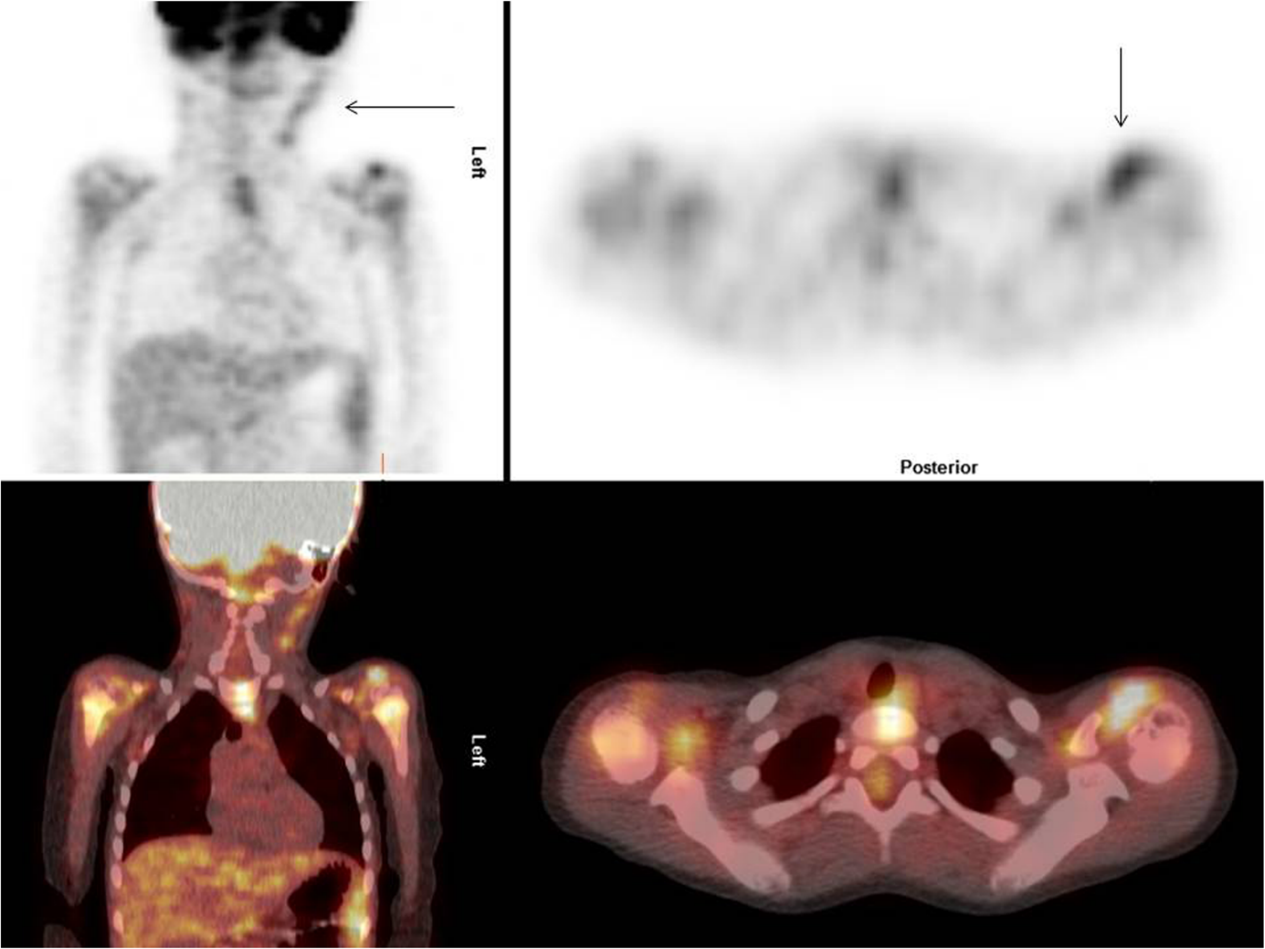
Detailed images of fludeoxyglucose positron emission tomography-computed tomography scan showing metabolically active cervical lymph nodes and increased fludeoxyglucose uptake in the left shoulder joint (*arrows*)

Increased FDG uptake was observed in multiple large joints, without profuse effusions on the low-dose CT (Fig. 1), and in multiple tendons, such as the tendons of the distal tibialis anterior and the ischiopubic ramus (Fig. 2). These findings suggested active polyarthritis and polytendinitis. Furthermore, the FDG-PET/CT showed multiple bilateral metabolically active cervical lymph nodes, probably due to a previous upper respiratory tract infection (Fig. 3). A diffuse, slightly increased FDG uptake in his spleen was observed, possibly in the context of an inflammatory response. A long, stretched FDG uptake in the medial part of his right lower leg was due to FDG stasis after intravenous injection at that site.

Subsequently, our patient was referred for ultrasound evaluation of all joints because of the discrepancy between the findings in the physical examination (no arthritis) and on the FDG-PET/CT (polyarthritis). This ultrasound examination (with power Doppler), however, did not show hydropses, synovitis, or other signs of arthritis in any joint. The dermatologist was involved during admission because our patient showed minimal petechiae in the popliteal area. No skin biopsy was performed at that time.

Treatment with diclofenac led to clinical improvement. Without establishing a diagnosis, our patient was discharged on diclofenac treatment thrice daily. Evaluation at our outpatient clinic the next week revealed the presence of multiple purpura on his lower limbs and buttocks. These skin lesions had been present for 1 day. A skin biopsy, taken from an area with active lesions on his lower leg, showed a leukocytoclastic vasculitis with positive IgA depositions, which confirmed the diagnosis of Henoch-Schönlein vasculitis.

The day after his visit to the outpatient clinic, our patient presented with bloody stools and abdominal pain. An abdominal ultrasound did not show any signs of invagination or thickened intestinal walls. His arthralgia and antalgic gait were still present. Owing to the severity of his clinical symptoms (pain and bloody stools), treatment with prednisolone was started and diclofenac was discontinued. Results of a urine evaluation at that time were normal. Our patient improved rapidly after starting prednisolone treatment; the dosage was therefore lowered 10 days later. After 15 days of treatment, our patient was free of symptoms. One month later a relapse in joint pain and abdominal pain occurred and he developed renal manifestations (hematuria and proteinuria). The dosage of the prednisolone was increased again. Finally, prednisolone treatment was stopped 2.5 months after starting, when our patient was completely free of symptoms.

## Discussion

The presented case demonstrates that FDG-PET/CT can show signs of inflammation in joints and tendons without signs of arthritis on physical and ultrasound evaluation in a pediatric patient with Henoch-Schönlein vasculitis. Approximately 80 % of patients with Henoch-Schönlein vasculitis develop arthritis or arthralgia. The exact etiology of arthritis in Henoch-Schönlein vasculitis is unknown. A study on synovial histology in Behçet’s disease, another systemic vasculitis, showed inflammation and infiltration with lymphocytes and neutrophils and no local signs of vasculitis [14]. The hypothesis of infiltration with lymphocytes could explain the increased FDG uptake in FDG-PET/CT in the presented case, because these activated lymphocytes consume FDG. In some cases, patients present with arthritis or arthralgia in the absence of the typical skin lesions [2, 7]. The absence of skin lesions can impede establishment of the diagnosis, as was illustrated in our case. Hypothetically, the treatment with IVIG in our patient may have delayed the inflammatory cascade, consequently leading to the delayed presentation of skin lesions [15]. It is well known that systemic symptoms may precede purpura in patients with Henoch-Schönlein vasculitis. The only case published on FDG-PET/CT in an adult patient with Henoch-Schönlein vasculitis describes the presence of a lung abscess [16]. However, as far as we know, the finding that FDG-PET/CT can reveal metabolically active, otherwise undetectable arthritis in patients with Henoch-Schönlein vasculitis has not previously been published. We suggest that FDG-PET/CT can detect increased inflammatory activity in patients with arthralgia before the clinical signs of arthritis are present.

This case illustrates that FDG-PET/CT can provide clues in a challenging diagnostic workup of a patient with unresolved arthralgia and fever of unknown origin. The finding of multiple bilateral active cervical lymph nodes suggests a preceding upper respiratory tract infection, a known trigger for Henoch-Schönlein vasculitis. It is well known that FDG-PET/CT is a very sensitive diagnostic tool to detect inflammation, especially in patients with fever of unknown origin and in patients with active rheumatic diseases [17, 18]. Available data suggest that FDG-PET/CT is favorable to MRI and CT as a diagnostic tool in patients with fever of unknown origin [17]. Furthermore, FDG-PET/CT appears to be superior to ultrasound and MRI in the diagnosis of large vessel vasculitis [18]. Studies on the role of FDG-PET/CT in small vessel vasculitis, like Henoch-Schönlein vasculitis, are lacking [19–21]. A recent study in patients with granulomatosis with polyangiitis, another small vessel vasculitis, showed that FDG-PET/CT can detect occult sites of disease activity [22]. The presented case suggests that FDG-PET/CT is also capable of detecting synovitis as a systemic manifestation of Henoch-Schönlein vasculitis. More studies are needed to evaluate the clinical use of FDG-PET/CT in Henoch-Schönlein and other small vessel vasculitides.

A disadvantage of FDG-PET/CT in children is the use of ionizing radiation. Because children are more susceptible to ionizing radiation than adults, there is a preference to use imaging techniques that do not use ionizing radiation, such as ultrasound or MRI. In specific cases, however, these techniques are not able to point to the diagnosis. In such cases the use of FDG-PET/CT in children is justified and can be very helpful and necessary to visualize inflammatory processes or other diseases showing a high metabolism, such as neoplasms. It must be emphasized that the most recent PET scanners with novel detector technology are capable of detecting ultra-low tracer doses, making reductions in injected dose possible. The recent introduction of hybrid PET-MRI systems can further reduce radiation burden.

## Conclusions

FDG-PET/CT might be able to detect subclinical synovitis in a patient with Henoch-Schönlein vasculitis. More research is needed to evaluate the clinical use of FDG-PET/CT in the diagnostic workup of patients with Henoch-Schönlein vasculitis.
